# Diseases of the Blood and Nutrition and Infectious Diseases

**Published:** 1887-03

**Authors:** 


DISEASES OF THE BLOOD AND NUTRITION AND INFECTIOUS DISEASES, being Vol. IV. of "A Handbook of Practical Medicine," by Dr. Hermann Eichhorst, and Vol. XII. of Wood's Library for 1886 (completing the set; price of set, $15.00). Illustrated. New York: William Wood & Company.

This volume contains sections eight, nine and ten of this valuable handbook of practical medicine. Each section is divided into a number of separate parts representing different diseases. Section eight is devoted to diseases of the blood and spleen, section nine to diseases of nutrition, while section ten, which forms the greater part of the work, is devoted to infectious diseases. The author includes under this head both infectious diseases, with typical local manifestations, such as measles, small-pox, scarlet fever, etc., and those which have variable local symptoms, such as tuberculosis, syphilis, leprosy, diphtheria, etc.

The work will prove a valuable addition to the library of any physician.

